# Use of Sorption of Copper Cations by Clinoptilolite for Wastewater Treatment

**DOI:** 10.3390/ijerph15071364

**Published:** 2018-06-28

**Authors:** Iveta Pandová, Anton Panda, Jan Valíček, Marta Harničárová, Milena Kušnerová, Zuzana Palková

**Affiliations:** 1 Faculty of Manufacturing Technologies with a Seat in Prešov, Technical University of Košice, Bayerova 1, 080 01 Prešov, Slovak Republic; anton.panda@tuke.sk; 2 Slovak University of Agriculture in Nitra, Technical Faculty, Tr. A. Hlinku 2, 949 76 Nitra, Slovakia; jan.valicek@vsb.cz (J.V.); marta.harnicarova@vsb.cz (M.H.); zuzana.palkova@uniag.sk (Z.P.); 3 Department of Mechanical Engineering, Faculty of Technology, Institute of Technology and Business in České Budějovice, Okružní 10, 370 01 České Budějovice, Czech Republic; milena.kusnerova@vsb.cz

**Keywords:** wastewater, environment, copper cations, sorption, zeolites

## Abstract

This paper from the field of environmental chemistry offers an innovative use of sorbents in the treatment of waste industrial water. Various industrial activities, especially the use of technological fluids in machining, surface treatment of materials, ore extraction, pesticide use in agriculture, etc., create wastewater containing dangerous metals that cause serious health problems. This paper presents the results of studies of the natural zeolite clinoptilolite as a sorbent of copper cations. These results provide the measurement of the sorption kinetics as well as the observed parameters of sorption of copper cations from the aquatic environment to the clinoptilolite from a promising Slovak site. The effectiveness of the natural sorbent is also compared with that of certain known synthetic sorbents.

## 1. Introduction

Heavy metals and their compounds endanger the environment as no method yet exists which provides a natural method for their decomposition. The predominant instruments for EU legislation which regulates pollution reduction of the aquatic environment is Council Directive 76/464/EEC on pollution, which was precipitated by certain dangerous substances discharged into the aquatic environment, as well as Council Directive 98/83/EC, which refers to the quality of drinking water. In wastewater treatment plants, heavy metal ions are removed through a number of methods, including oxidation-reduction reactions, by coagulation and sedimentation, magnetic separation, activated carbon sorption, ion exchange within ion exchangers, and biochemical methods which use algae [1,2,3].

Currently, research is focused on three areas: (1) the application of natural and modified zeolite composites, (2) the use of natural materials that are more affordable than synthetic ones, and (3) the reduction of metal in water [3,4,5]. Copper is an essential element found in metalloenzymes, but it affects living organisms toxically at higher concentrations. Therefore, it is important to monitor the concentration of this element in water and, in cases of increased concentrations, its necessary adjustment. Conventional chemical methods can be costly, as they require the use of several chemicals; a further disadvantage is the production of waste by-products. Therefore, it is preferable to attach copper in the form of its cations to sorbents. Several types of synthetic zeolites are suitable for this purpose; however, their cost is disadvantageous in comparison with natural zeolite. Their sorption and ion exchange capabilities, which derive from their inherent structure, are interesting for the purposes of cleaning water. An uptake of zinc (Zn), copper (Cu), and lead (Pb) from aqueous solutions by ion exchange on natural zeolitic tuff has been studied. The Croatian zeolite clinoptilolite from the Donje Jesenje deposit has been used as a natural ion exchanger. The efficiency of removal is higher for Pb and Cu than for Zn ions [5].

According to the literature, the sorption method used in water treatment processes has recently become a dominant method, especially in the use of locally available, economically undemanding, and natural materials. Such materials include natural zeolites, which, because of their chemical composition, represent inorganic alumino silicate cation exchangers. As a carrier matrix, zeolite has the necessary component to meet the demanding criteria to produce new composite materials. In contrast to amorphous organic ion exchangers, natural zeolites have a solid skeleton comprising silicon and aluminium polyoxides. Additionally, natural zeolites have a sufficiently large adsorption surface and are hydrophilic, polar, micro-porous, semi-resistant to thermal and radiation effects, affordable, and exhibit lower abrasive properties than activated carbon, which predisposes them to an appropriate hydrodynamic use in practice [6,7].

The three-dimensional zeolite structure consists of a regular arrangement of tetrahedral [SiO_4_]^−4^ and [AlO_4_]^−5^. In the [SiO_4_]^−4^ tetrahedra network, part of the silicon atoms is replaced isomorphically by aluminum atoms. The negative charge of the formed zeolitic grid is compensated by cations that are located in extra-grid positions. These are most often cations of the alkali metals and alkaline earth metals [6,8,9,10,11,12,13,14,15,16,17,18,19,20,21]. These cations can be replaced by other cations with the use of sorption and ion exchange. Natural zeolites are hydrated alumino-silicates, which are characterized by their ability to sorb heavy metal cations from aqueous solutions. Cations are immobilized on the zeolite by two mechanisms: ion exchange and chemisorption [22]. In this way, as in other micro-porous materials, the ion exchange sorption between the components of the liquid and solid phases is carried out according to the surface diffusion and the internal diffusion mechanism.

The application possibilities of natural zeolites ensue from their specific physicochemical properties, such as ion exchange, sorption, and the molecular sieve properties derived therefrom. Concurrently, the properties of natural zeolites offer possibilities of dehydration and hydration as well as the silicate structure itself, not to mention the micron dimensions of crystals with highly active specific surface areas. In terms of practical use, clinoptilolite deposits offer numerous interesting potential uses, for example, in the purification of gases, in the treatment of contaminated waters, and in agriculture regarding the neutralization of acidic soils. The ability of ion exchange and adsorption properties implicate a use of natural zeolites as effective carriers of herbicides, pesticides, as well as mineral fertilizers. After thermal activation, clinoptilolite has a relatively high sorption capacity for several gases, such as NH_3_, CO_2_, H_2_S, SO_2_, and NO_x_ [23]. In the United States and Japan, several water treatment stations use clinoptilolite for the protection of drinking water sources. The use of natural clinoptilolite for the removal of ammonium ions from water is also well known. After its regeneration by diluted sulphuric acid, an ammonium sulphate is obtained, which can be used as a fertilizer. In several locations around the world, clinoptilolite has demonstrated its ability to capture radioactive elements. In this respect, natural zeolites are also used for the purification of low-activity liquid wastes from nuclear power plants, predominantly for the capture of radioactive strontium and radioactive cesium. Zeolites have a high affinity for cesium. After the modification of natural clinoptilolite by the solution of cations of metals, clinoptilolite acquires catalytic properties and, accordingly, is used for the purification of gases [24].

The latest water treatment technologies comprise sorption on a solid layer. The sorption isotherm and the kinetic evolution of sorption must be known for the design of the cleaning process arrangement. This article represents a laboratory experiment for the determination of sorption parameters of sorption of cupric cations on natural zeolite-clinoptilolite, which is a low-cost and environmentally friendly sorption material. Experimental data obtained from equilibrium tests were analyzed using the Freundlich model.

## 2. Materials and Methods

### 2.1. Properties of Used Clinoptilolite

We tested the natural zeolite clinoptilolite, which was acquired at the Nižný Hrabovec site, to investigate the possibility of replaceing synthetic zeolites with natural ones. This Slovak deposit is economically significant, with annual mining output ranging from 40 to 50 thousand tons. Reserves of approximately 9,500,000 tons ensure the long-term availability of this natural material.

Clinoptilolite is a potassium—calcium type mineral with a pore size of 0.3–0.4 nm. Clinoptilolite composition is expressed by the formula [8]: (Na, K)_4_ Ca (Al_6_ Si_30_ O_72_) × 24 H_2_O.

Chemical and physical properties of the clinoptilolite are presented in Table 1, Table 2 and Table 3.

We compared this natural material with several types of synthetic zeolites with respect to the cupric cation sorption, specifically, with nalsite, calcite, and y-site. Nalsite is a synthetic zeolite of the type 4 A with a pore size of 0.4 nm. Its chemical composition in a dehydrated state is expressed by the formula: Na_2_O·Al_2_O_3_∙2 SiO_2_. Calsite is a synthetic zeolite of the type 5 A with a pore size of 0.5 nm. Its chemical composition in the dehydrated state is expressed by the formula: CaO∙Na_2_O∙Al_2_O_3_∙2 SiO_2_. Y-site is a synthetic zeolite of the Y type with a pore size of 0.9 nm. Its chemical composition in the dehydrated state is expressed by the formula: Na_2_O∙Al_2_O_3_∙4.5SiO_2_ [12,14]. Synthetic zeolites are at low pH (pH ≤ 3) subjected to hydrolysis and elution of alumina from the skeleton, offering a use case for natural clinoptilolite in industrial practice.

For a quantitative determination of the concentration of copper cations in aqueous solution at precise time intervals, absorption photometry was applied with the use of the equipment Optima DIGITAL COLORIMETER Model AC 114 (Optima, Tokyo, Japan). Measurements were realized at a wavelength of 620 nm using the calibration curve method. For plotting the calibration curve, we used a set of standard solutions with the concentration of copper cations ranging from 2.50 to 9.05 g·dm^−3^. After addition of ammonia with ammonium ion, the copper produced a blue-violet complex composition [Cu(NH_3_)_4_]^2+^. For determination of the absorption maximum of this complex, we plotted the graphical dependence A = f (ʎ) using a standard solution. Accordingly, we determined the adsorption maximum for the wavelength ʎ of 620 nm. The analytical measurement of concentration of copper cations was based on the construction of the calibration curve in dependence on the absorbance from the concentration of the standard A = f (c).

Determination of dependence of the sorbed quantity on the sample concentration in the solution was performed by the method of the container experiments. To the weighed samples of sorbent (natural clinoptilolite) of 50 g, the same volume (0.1 dm^3^) of an aqueous solution of copper cations was added. For this model, we used samples with an initial mass concentration of 2.56 g·dm^−3^, 4.95 g·dm^−3^, 6.72 g·dm^−3^, 7.05 g·dm^−3^, and 8.55 g·dm^−3^. The samples were mixed and, at regular one-hour intervals, the concentration of copper cations was measured photometrically in the aken liquid phase. With the liquid phase, we also took the equivalent amount of the sorbent. On the basis of the measured values, the sorbed quantities were calculated for the individual time intervals. The measurements were performed until achievement of the equilibrium state, i.e., the state when the concentration of copper cations in the solutions ceased to change. The experiment was performed three times, with a standard deviation of 1.0049 calculated for the measured equilibrium concentrations.

### 2.2. Sorption of Cupric Cations from Aquatic Solutions

The process of sorption of a chemical substance from solution to solid matter can be expressed as a result of the reversible reaction, sorption and desorption, which achieves the resulting equilibrium between the concentrations of the chemical substance in both phases. This process is studied by evaluating the equilibrium concentration of a chemical substance in the sorbent as a function of the total equilibrium concentration in solution at a given temperature. This dependence is expressed by isotherms. The efficiency of the sorption of soluble matters on the solid matrix to the aqueous solution is most often expressed by the effective distribution coefficient *K_R_*, which is the slope of a straight line of the linear sorption isotherm and which gives the share of the sorbed amount of the substance in the solid phase (*c_s_*) to its equilibrium concentration in the solution (*c_r_*) during the equilibrium state [26,27]. This parameter is a quantitative indicator of substance distribution between the solid and liquid phases *K_R_* = *c_s_*/*c_r_* [28,29]. The amount of the sorbed substance per sorbent unit increases linearly with the increasing concentration at low surface coverage, under three assumptions that must be met. The sorption energy must be the same for all sorption sites and it must be independent of the degree of coverage; sorption should take place only at localized sorption sites and without interaction between the sorbed molecules, the sorption capacity being a one-layer coating [30].

Assuming that the sorbed substance reaches the sorbent surface by molecular diffusion through a boundary diffusion layer, it is possible to generally express the concentration of the sorbed substance *c* at a time *t* by the Equation (1) [30].

(1) $$c\;=\;\frac{\lambda \cdot c_{r}-\beta \cdot e^{\bar{\rho }\cdot t}}{\lambda -e^{-\bar{\rho }\cdot t}}$$

where *λ*, *β*, *ρ* are constants that are obtained from the measured values of concentration for individual time intervals (“β” and “*λ”* have a concentration dimension, “*ρ”* has a dimension of reciprocal value of time, *c*_0_ is the initial concentration of copper cations in solution, *c_r_* is the equilibrium concentration of copper cations in the solution).

(2) $$\lambda \;=\;\frac{c_{0}-\beta }{c_{0}-c_{r}}$$

where:

(3) $$\beta =\frac{2\cdot \gamma \cdot c_{1}-c_{0}-c_{2}}{\gamma -1}-c_{r}$$

(4) $$\gamma =\frac{\left(c_{0}-c_{r}\right)\cdot \left(c_{2}-c_{r}\right)}{{\left(c_{1}-c_{r}\right)}^{2}}$$

(5) $$\bar{\rho }=\frac{1}{t}\cdot \ln\frac{\left(c_{0}-c_{r}\right)\cdot \left(c-\beta \right)}{\left(c_{0}-\beta \right)\cdot \left(c-c_{r}\right)}$$

## 3. Results and Discussion

### Measurement Results and Their Evaluation

**Figure 1:**
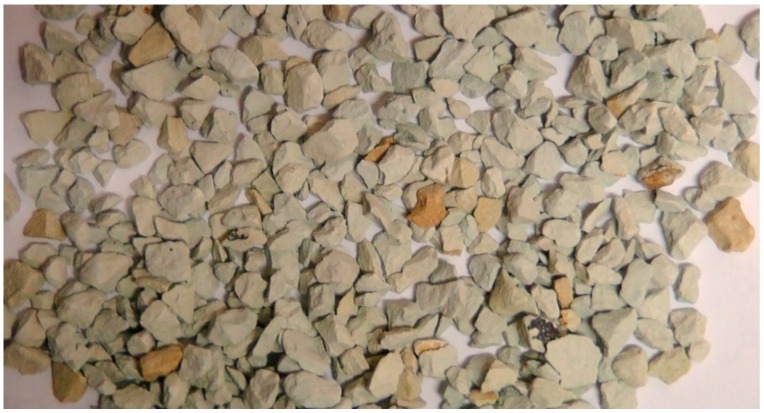
Zeolite clinoptilolite with a grain size from 2.5 to 5 mm.

Laboratory measurements were focused on the sorption of cupric cations from the aquatic environment. The kinetic course of sorption of cupric cations on natural sorbent-clinoptilolite with a grain size of 2.5–5 mm (Figure 1) and on synthetic zeolites was investigated. From the measured concentration values, the efficiency of the individual sorbents was calculated. On the basis of the measured concentrations of cupric cations in solution, the effective distribution coefficients were calculated for individual sorbents. The degree of cleaning of the contaminated water was evaluated using the sorption efficiency parameter expressed as a percentage.

In individual types of sorbents, the influence of duration of contact on sorption of cupric cations was recorded within 48 h. To define the time required to achieve chemical equilibrium, a dependence of the sorbent quantity on the duration of contact of the sorbent with sorbate was investigated. Individual types of sorbents weighing 50 g were used for the experiments. The sorbents were exposed to an aqueous solution of cupric cations with a volume of 0.25 dm^3^ with an initial concentration of 2.54 g·dm^−3^. In individual samples of sorbents, samples of solutions were taken at the exact time intervals until equilibrium was reached for analytical determination of the content of cupric cations. On the basis of the analysis, the sorption evolution with the use of individual sorbents was established (Table 4).

The measured results show that, as a result of the sorption of cupric cation, the most rapid reduction of cations was on the nalsite, where the sorption capacity had been exhausted during the first hour. In terms of sorption rate, the second most rapid reduction was calsite, in which the reduction in concentration to almost zero was recorded after 120 min. Y-site was the third most rapid according to the sorption rate. Clinoptilolite, with sorption equilibrium for this sorbent, was reached after 48 h and showed the slowest sorption rate. The highest efficacy was recorded both in nalsite, which, after 60 min, showed 100% efficiency, and in calsite, with a reduction of cupric cations to 5% of the original concentration after one hour. Total efficacy at steady state was 94%. The efficiency of the y-site at steady state was 92%, and, after the first hour, the concentration of cupric cations was reduced to 13% of the original concentration. On the natural zeolite clinoptilolite, after the first hour, the cupric cations fell to 60% of the original concentration; the efficiency after 48 h was 81%. The efficacy was calculated by the formula *η* = (*c_1_* − *c_2_*/*c_1_*)·100 where *c_1_* is the initial concentration of cupric cations in the solution and c_2_ is the concentration of cupric cations in steady-state solution.

The effective distribution coefficients recorded at steady state for the compared sorbents are presented in Table 5.

The results indicate the advantage of synthetic zeolites in comparison with natural clinoptilolite according to their faster evolution of sorption and higher efficiency. In contrast, the natural zeolite, due to its rich deposits, is more affordable. As a comparison, we provide the relative prices of individual sorbents: clinoptilolite-0.078 euro/kg, nalsite-4.813 euro/kg, calsite-5.411 euro/kg, and y-site-9.892 euro/kg. On the basis of these facts, we focused our subsequent experiments on clinoptilolite.

In the case of clinoptilolite, the constants were calculated from the measured concentration values for individual time intervals and were then used for the searched relationship *c* = *f* (*t*). The values required for the calculation of the constants for the Equation (1) as well as the calculated values of the concentration of cupric cations in the solution are provided in Table 6.

For the calculation of the constants, an average value *ρ_p_* = 0.0053 was used. The searched relation *c* = *f* (*t*) was according to the Equation (1) with the use of the calculated constants according to the following

(6) $$c_{v}=\frac{0.508\cdot 1.234-0.02\cdot e^{-0.0053\cdot t}}{1.234-e^{-0.0053\cdot t}}$$

Standard deviation calculated according to the relation [27,30,31,32,33,34] had the value of 0.6.

(7) $$S=\frac{\sqrt{\sum {\left(c_{m}-c_{v}\right)}^{2}}}{n-2}$$

**Figure 2:**
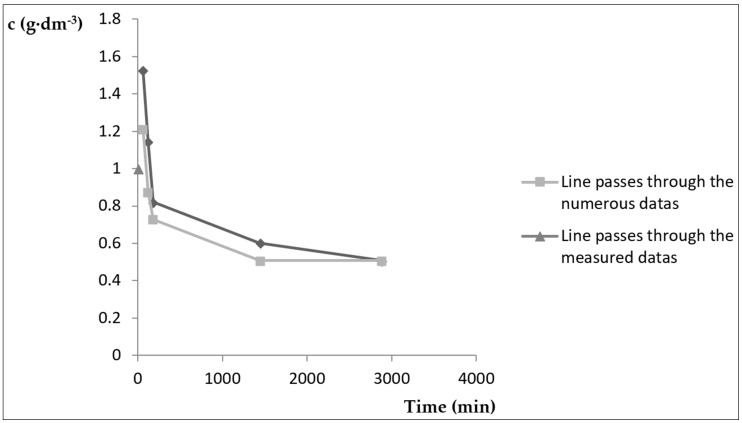
Dependence of the weight concentration c of cupric cations in solution according to time.

Figure 2 shows the experimentally determined values and the curve fitted to them, for which the parameters were calculated according to the Equation (6).

Further laboratory measurements were aimed at the evolution of the adsorption isotherm of sorption of cupric cations on the natural zeolite clinoptilolite with a grain size of 2.5–5 mm from Nižný Hrabovec. For the sorption of cuprous cations, we used model solutions with an initial weight concentration of *c*_0_ 2.56 g·dm^−3^, 4.95 g·dm^−3^, 6.72 g·dm^−3^, 7.05 g·dm^−3^, and 8.55 g·dm^−3^. For determination of the time required to achieve balance in the system, we monitored the dependence of the sorbed quantity from the moment of contact of the sorbent with the adsorbate at a temperature of 25 °C. The results were processed graphically and mathematically with the use of the Freundlich adsorption isotherm. The evolution of sorption for all model samples was monitored at precise one-hour time intervals.

With prolonged contact time of the sorbent with the solution, the concentration of the cupric cations in solution asymptotically approached the equilibrium concentration c_r_. The quantity of absorbed cupric cations was calculated according to the Equation (8) [27,31,32,33] as the difference between the initial concentration of c_0_ and the concentration in solution in the equilibrium state *c_r_*, where a is the sorption capacity [mg·g^−1^], *V* is the volume of the solution, and m is the sorbent mass.

(8) $$a=\frac{c_{0}-c_{r}}{m}\cdot V$$

To analyze the equilibrium experimental data for adsorption, the Langmuir or Freundlich isothermal models were used. The Langmuir isothermal model is based on the assumption that the surface areas of the adsorbent are homogeneous, and that the maximum adsorption is limited to covering the surface of the monolayer; in contrast, the Freundlich isothermal model is based on the assumption of heterogeneous surface areas and multilayer surface coverage. If the dependence *a* = *f* (*c_r_*) can be expressed by the equation

(9) $$a=\frac{a_{m}\cdot b\cdot c_{r}}{1+b\cdot c_{r}}$$

The experimentally obtained values were plotted with the coordinates X_i_ = c_r,i_, Y_i_ = c_r,i/_a_i_ to create a line. The Freundlich isotherm assumes that the adsorbate concentration on the surface of the adsorbent increases with the increase in adsorbate concentration. This isotherm is based on sorption on a heterogeneous surface, which is expressed by an exponential equation [9,30].

If the Freundlich isotherm satisfies the expression of the dependence *a* = *f* (*c_r_*), it is possible to fit the line with the experimental values plotted in the coordinates X = log *c_r_*, Y = log *a*. From the obtained values of the angular coefficient of this line and from the section of the line on the Y-axis, it is possible to calculate the sought constants of the Freundlich isotherm. The experimentally obtained and calculated parameters are presented in Table 7.

The experimentally measured and computed values plotted at coordinates X = c_r,i_, Y = c_r,i_/a_i_ did not conform to a straight line. For this reason, it was not possible to describe the dependence a = f(c_r_) by the Langmuir equation. We constructed the straight line after having calculated the logarithm of the quantities a and c_r_ and their plotting in coordinates X = log c_r_, Y = log a. For the calculation of the searched quantities, we adopted the Freundlich model.

**Figure 3:**
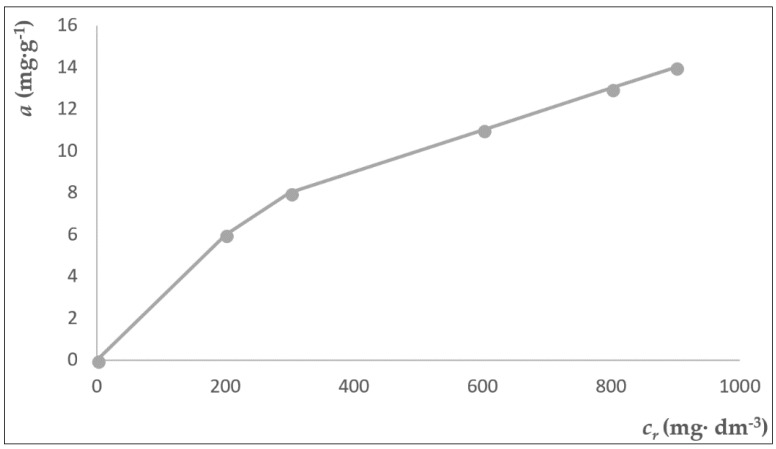
Sorption isotherm for sorption of cupric cations from solutions to clinoptilolite.

Assuming an adsorption on a non-homogeneous adsorption surface, a Freundlich adsorption isotherm was used with the following form [9,27,32,33,34,35,36,37,38]:

(10) $$a=K\cdot c_{r}^{1/n}$$

where *K* and *n* are Freundlich constants, which indicate the adsorption capacity of the adsorbent and the adsorbent adsorbate affinity. Graphical representation of the Freundlich adsorption isotherm is shown in Figure 3.

The constant values of the isotherm are determined by the least squares method and from the linearised Freundlich equation:

(11) $$\log a=\log K+\frac{1}{n}\cdot \log c_{r}$$

**Figure 4:**
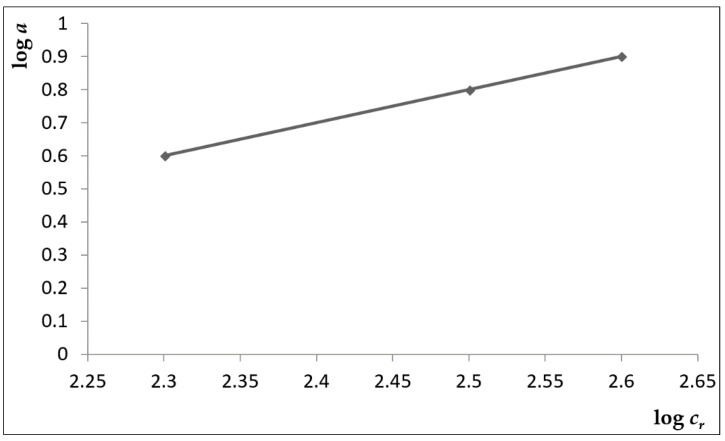
Logarithmic shape of the Freundlich isotherm.

The logarithmic shape of the isotherm is shown in Figure 4.

The constant values of the isotherm are determined by the least squares method. By this method, we calculated for the constant, *K,* a value of 5.012 and for *q* a value of 0.67.

*n* = 1/*k* = 0.23

*K* = 10^q^ = 10^0.67^ = 5.012

Accordingly, the isotherm shown in Figure 2 can be expressed by the equation

*a* = 5.012 ∙ *C_r_*^1/0.23^.

The Freundlich isotherm [3] assumes that the adsorbate concentration on the surface of the adsorbent increases with an increase in the concentration of solution. Our experiment confirms this. This finding is consistent with the results of the experiments performed by the cited authors. An increase in a concentration generally results in an increase in the amount of copper adsorbed and the rate of adsorption. According to the results obtained by Zendelska et al., the adsorption capacity will increase with an increase in initial concentration until the system reaches a saturation point [38].

Because dependence of the sorbed quantity on the equilibrium concentrations in the logarithmic form was linear, the measured sorption isotherm conformed to the Freundlich sorption isotherm.

Adsorption isotherms tested under laboratory conditions can be used for preliminary investigation as to the potential technological use of natural zeolite in the sewage water treatment process. The isotherm parameters were calculated with the use of experimental results of the sorbed copper cations per gram of sorbent versus their equilibrium concentrations in solution. We compared the sorption capacity calculated for the sample of the natural zeolite used in our experiment with the sorption capacity determined in previous experiments [39]. In the case of monoionic form of sodium, a sample of approximately the same initial concentration, the sorption capacity of the unmodified sample reached half the capacity of the modified sample. When a natural sample was used, we reached equilibrium after 24 h; in contrast, with the modified sample, we reached equilibrium in three hours.

Peric et al. [5] observed the sorption behaviour of Zn, Cu, and Pb on the natural zeolite clinoptilolite from Croatia. Their results show that ion exchange capacity for Cu and Pb is twice the size of Zn under the same experimental conditions, when equilibrium for Cu was achieved after 72 h. In light of these facts and the results obtained, the sorption-ion-exchange method using the natural clinoptilolite is an efficient process for removing heavy metal ions from wastewater containing lower concentrations of contamination. To accelerate this process, it is appropriate to modify the natural clinoptilolite, for example, into the Na-form. According to the results obtained by Holub et al. [40], the sorption of copper cations can also be influenced by the sorbent grain size. More favourable results were obtained with the use of natural clinoptilolite of smaller granularity. Butnariu et al. [41] observed the effects of the sorption of environmental applications by various source materials of natural organic matter. The results suggest a potential for obtaining efficient and cost-effective engineered natural organic sorbents for environmental applications. 

## 4. Conclusions

In emergency situations, the principle of chemical reactions, such as precipitation, can be used for immediate reduction of concentrations of cations of heavy and toxic metallic elements in water. However, it is required that the elimination of ongoing and prolonged contamination is performed by inexpensive concentration-reducing methods. This creates an opportunity for the use of natural zeolites. Although these have a lower sorption rate in comparison with synthetical zeolites, they are much cheaper. The Zeolite-based sorption technology does not require significant space or the use of expensive chemicals. Because this is a natural and easily accessible material, it is assumed that this method could be used in the future in greater extent for cleaning water from cupric cations and from other heavy metals in process plants where high rates of cleaning are not required.

On the basis of the above results, the ability of adsorbent based on the natural zeolite to remove cupric cations from the aqueous environment was confirmed, while sorption capacity of the sorbent increased with the initial concentration of cupric cations in the aqueous solution. We described the evolution of the sorption process by the Freundlich isotherm.

The contact filtration through a suitable material represents an economically acceptable and undemanding technology for removing cupric cations from water. The acquired findings of basic research on specific natural sorbents can, in perspective, provide an important information for technological processes of water purification. The use of sorbents in water purification processes can help the efforts to increase water reserves through a safe re-use of wastewater.

## Figures and Tables

**Table 1:** Clinoptilolite chemical composition [25].

Compound	Content	Compound	Content
SiO_2_	65–71.3%	Fe_2_O_3_	0.7–1.9%
Al_2_O_3_	11.5–13.1%	MgO	0.6–1.2%
CaO	2.7–5.2%	Na_2_O	0.2–1.3%
K_2_O	2.2–3.4%	TiO_2_	0.1–1.3%
P_2_O_5_	0.02%	Si/Al	4.5–5.4%

**Table 2:** Ion exchange properties [25].

Cation	Overall Interchange [mol·kg^−1^]
Ca^+2^	0.64–0.98
Mg^+2^	0.06–0.19
K^+^	0.22–0.45
Na^+^	0.01–0.19

**Table 3:** Physical properties [25].

Physical Property	Value
Temperature of softening	1 260 °C
Temperature of fusion	1 340 °C
Stability in acids	79.50 °C
Density	2200–2440 kg·m^−3^

**Table 4:** The process of sorption in sorbents.

Time (h)	Clinoptilolite	Sorbed Amount (g·dm^−3)^	Y-Site	Nalsite
Sorben		Calsite		
1	1.03	2.42	2.22	2.54
2	1.42	2.53	2.29	0
3	1.73	0	2.38	
24	1.96		0	
48	2.2			
72	2.2			

**Table 5:** Effective distribution coefficients calculated for various types of sorbents in the concentration of cupric cations of 2.54 g·dm^−3^.

Type of Zeolite	*c*_s_	*c*_r_	*K_R_*
Calsite	2.40	0.137	17.5
Y-site	2.35	0.182	5.5
Nalsite	2.54	0	2.54
Clinoptilolite	2.03	0.508	4.0

**Table 6:** Temporal change of cupric cations and calculated parameters.

*t* (min)	*c_m_* (g·dm^−3^)	*σ* (min^−1^)	*c_v_* (g·dm^−3^)
60	1.524	0.00301	1.21
120	1.143	0.0029	0.874
180	0.82	0.0039	0.729
1440	0.601	0.0112	0.508
2880	0.508		0.508

**Table 7:** Obtained experimental parameters.

*c_r_* (mg·dm^−3^)	*a* (mg∙g^−1^)	log c*_r_*	log *a*	*c_0_* (g·dm^−3^)
200	4.9	2.3	0.6148	2.56
300	6.18	2.47	0.79	4.95
700	11	2.8	1.04	6.72
800	12	2.9	1.079	7.05
820	15.7	3.07	1.2	8.55
